# A retrospective cohort study of autogenous iliac strut bone grafting in large bone defects of the lower extremity

**DOI:** 10.1038/s41598-024-56726-7

**Published:** 2024-03-13

**Authors:** Incheol Kook, Jooyoung You, Dong Hong Kim, Ki-Chul Park, Kyu Tae Hwang

**Affiliations:** 1 Department of Orthopaedic Surgery, Hanyang University Hospital, 222 Wangsimni-ro, Seongdong-gu, Seoul, 04763 Republic of Korea; 2 Department of Orthopaedic Surgery, Hanyang University Guri Hospital, Guri, Gyeonggi-do Republic of Korea

**Keywords:** Trauma, Outcomes research

## Abstract

Autogenous iliac bone graft (AIBG) is the treatment of choice for managing bone defects, and favorable results have been reported for bone defects < 5 cm in length. In large bone defects ≥ 5 cm, it is difficult to obtain good results with simple bone grafting, and other management options have drawbacks, such as long immobilization periods and high complication rates. We hypothesized that AIBG in the strut form might show favorable results in large bone defects with minimal complications. This study aimed to investigate the outcomes of strut-type AIBG and evaluate its effectiveness compared to cancellous AIBG. This retrospective study included 50 patients who underwent AIBG for bone defects at a single institution between March 2011 and April 2020. We performed corticocancellous AIBG in a strut form to manage bone defects ≥ 5 cm in the lower extremities. The strut bone was harvested along the iliac crest and grafted slightly longer than the bone defect to apply a sufficient compressive force. Demographic information and radiographic and clinical results of patients who underwent strut AIBG (Group S) were analyzed. The outcomes of union, time to union, complications, and reoperation were compared with those of patients who underwent cancellous AIBG (Group C). The study population comprised 37 men (74%) and 13 women (26%), with a mean age of 50.0 (range: 19–78). The average follow-up period was 25.6 months (12–104 months). Group S included 16 patients with a mean bone defect length of 6.8 ± 1.2 cm. In Group S, union was achieved in all patients, with an average time to union of 6.7 months. Complications occurred in four cases, all related to wound problems. Group C comprised d 34 patients with a mean defect length of 2.8 ± 1.1 cm. Complications occurred in five patients in Group C, including four soft tissue problems and one implant failure. When comparing the outcomes of Groups S and C, no significant differences were observed. AIBG is an effective and safe technique for managing bone defects. Strut AIBG can be used effectively for bone defects ≥ 5 cm in the lower extremities.

## Introduction

Bone defects of the lower extremities are commonly accompanied by acute trauma or may occur due to infections or nonunion, and an autogenous bone graft is recommended as the treatment of choice with good results^1^. Autogenous bone grafts provide combined mechanisms of osteoconduction, osteoinduction, and osteogenesis and have the advantage of a high union rate without the risk of immune response^1,2^. Despite these advantages, the characteristics of autogenous bone grafts, such as limited harvest volume, difficulty in filling sufficient space as the grafted cancellous bone collapses, and failure to maintain length, preclude their use for large bone defects ≥ 5 cm in length^3^.

Previous studies on autogenous cancellous bone grafts have reported bone resorption or union failure when performed for large bone defects^4–8^. Methods such as bone transfer and vascularized fibular grafting have been proposed for the management of large bone defects, but they have the drawbacks of long immobilization periods and high complication rates^9^. Therefore, there is growing interest regarding alternative procedures that can address large bone defects ≥ 5 cm more effectively and less timeously.

Through a literature search, we found that non-vascularized fibular strut grafts for large bone defects after trauma or tumor resection and autogenous strut bone graft for anterior spinal fusion showed favorable results^10–12^. A strut bone graft is composed of corticocancellous bone and can maintain its length while providing volume to the bone defect. We hypothesized that autogenous bone grafts in the strut form might show favorable union rates in large bone defects ≥ 5 cm with minimal complications^13^. This study aimed to analyze the outcomes of the use of autogenous strut bone grafts for large bone defects ≥ 5 cm in the lower extremities and to compare them with those of autogenous cancellous bone grafts, which are accepted as the standard treatment for bone defects < 5 cm.

## Materials and methods

### Patient selection and ethical approval

Between March 2011 and April 2020, 61 consecutive patients with lower-extremity bone defects who underwent autogenous bone grafting at a single tertiary referral center were enrolled. The inclusion criteria were patients with bone defects in the long bones of the lower extremities, treated with autogenous iliac bone graft (AIBG). The exclusion criteria were follows: age < 18 years, bone defects caused by pathological fractures or tumor resection, and less than 12 months of follow-up. This retrospective study was performed in accordance with the Declaration of Helsinki and was approved by the Institutional Review Board of Hanyang University Hospital (IRB No: 2021-05-039). Requirement for informed consent was waived by the Institutional Review Board of Hanyang University Hospital due to the retrospective nature of the study.

Based on the type of grafted bone, the patients were classified into two groups: a strut bone graft group (Group S) and a cancellous bone graft group (Group C). We intended to compare outcomes after single-stage strut or cancellous bone grafting for bone defects ≥ 5 cm, but previous studies have reported poor outcomes after cancellous grafting for large bone defects, including low union rates and high complication rates^6–8^. Currently, cancellous bone grafting alone is not recommended for large bone defects, and it is recognized as the standard of care for smaller bone defects (< 5 cm)^1,3^. If cancellous bone grafting is performed on patients with large bone defects and these patients are included in Group C, it may not only harm the patient but also raise ethical issues for the study. Therefore, as an alternative, we set Group C as patients who received standard treatment (cancellous bone graft for bone defects < 5 cm) and compared the treatment effect of strut bone graft to that of standard treatment.

### Data collection and assessments

Bone union was determined by the disappearance of the fracture line or formation of a bridging callus on at least three cortices on plain anteroposterior (AP) and lateral radiographs. It was quantitatively assessed per the Radiographic Union Scale of Tibial Fractures (RUST)^14^. The RUST is based on the cortical callus and fracture line on AP and lateral radiographs, and scores are assigned to each of the four cortices, including 1 (no callus with visible fracture line), 2 (bridging callus, visible fracture line), or 3 (bridging callus, invisible fracture line), with a total score ranging from 4 (definite nonunion) to 12 (definite union). Union was defined by a RUST score ≥ 10^14^. The RUST score was assessed by two orthopedic surgeons who did not participate in the surgery, and discrepancies were resolved by consensus. Nonunion was defined as a fracture that did not heal without further intervention or failure to achieve bone union for 9 months after surgery with no progressive callus formation for 3 months on sequential radiographs^15,16^. Patients with suspected nonunion on plain radiographs were evaluated by computed tomography (CT) at 6 months or 1 year postoperatively. Nonunion was classified as hypertrophic, oligotrophic, or atrophic according to the classification system of Weber and Cech^17^. Complications related to bone union or soft tissue that occurred during admission and outpatient follow-up were also investigated. Donor site pain was defined as pain lasting more than 6 weeks after surgery^18^. The presence of hematoma formation was monitored by regular postoperative dressing changes. Infection was defined as an inflammatory response such as redness, heat, or swelling around the donor site, and blood tests were performed to check white blood cell count, C-reactive protein (CRP), and erythrocyte sedimentation rate (ESR) levels. Sensory changes at the donor site were defined as symptoms lasting for more than 3 months postoperatively^19^. Reoperation was defined as all cases requiring secondary surgery because of nonunion or metal failure.

Demographic data, such as age, sex, diabetes status, and smoking status, and data regarding the follow-up period were investigated. Data on graft type (strut bone or cancellous bone); the cause (trauma, infection, or nonunion), location (femur or tibia), area (diaphysis or metaphysis), length and type (segmental or partial) of the bone defect; and open fractures were recorded. Regarding the causes of bone defects, infection was defined according to the fracture-related infection (FRI) consensus definition^20^, whereby an infected nonunion was also classified as an infection. The bone-defect length was measured in the operative field and determined as the longest length along the longitudinal axis of the bone defect. The volume of the bone defect was also estimated using pre- and post-operative CT. Bone defect type was defined as segmental if 100% of the circumference was lost and partial if more than 50% was lost while having cortical continuity. Open fractures were classified based on the Gustilo-Anderson classification (G-A)^21,22^. The fixation methods, flap surgery, and timing of the flap (before or simultaneously with the bone graft) were also investigated. Plates, nails, or both were used for fixation, and it was performed at the discretion of the attending surgeon. In nonunion cases, bone grafting was performed without additional fixation when mechanical stability was considered sufficient.

### Surgical technique for autogenous iliac bone graft

**Figure 1:**
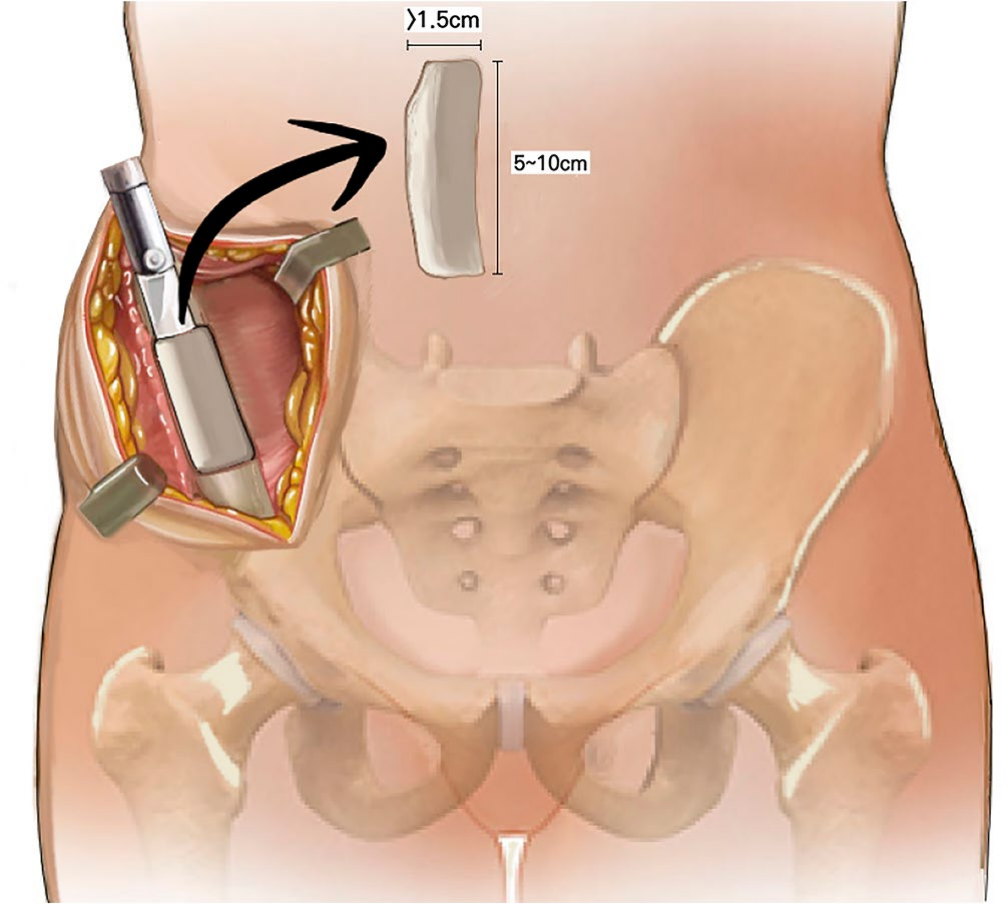
Schematic illustration of the autogenous iliac strut bone harvesting technique. A longitudinal incision is made along the iliac crest, extending 3 cm posterior to the anterior superior iliac spine (ASIS). A fissure is made in the cortex of the iliac crest with a mallet and straight osteotome, and the cortex of the inner and outer table is outlined with a curved osteotome. The corners were made perpendicular and did not intersect each other. The strut bone graft was harvested to have a length of 5–10 cm and a depth of > 1.5 cm. The sufficient depth not only enhanced the stability of the graft, but also increased the osteogenic property by ensuring that enough cancellous bone was contained within the graft.

All bone grafts were harvested along the anterior iliac crest using the anterior approach. The patient was placed supine on the operating table, and a bump was placed under the pelvis. A skin incision was made along the anterior iliac crest, the subcutaneous tissues were bluntly dissected, and the overlying muscles were stripped subperiosteally. The strut-type corticocancellous bone graft was harvested using an osteotome, and a cortical window was used to harvest the cancellous bone graft. Strut bone grafting was performed only when the bone defect length was ≥ 5 cm (Fig. 1). To prevent lateral femoral cutaneous nerve injury and iliac crest stress fracture, the skin incision, soft tissue dissection, and bone harvesting were stopped 3 cm posterior to the anterior superior iliac spine (ASIS)^23,24^. Other methods were used to prevent fractures at the donor site as well. Only the cortex of the iliac crest was gently struck with an osteotome, first creating a fissure all around the cortical surface, followed by a deeper strike to reach the cancellous portion^25^. In creating the fissures in the cortex, each edge was made perpendicular, being careful not to intersect. If the corners intersect or meet at an oblique angle, there is a risk of iatrogenic fractures at the corners during graft harvest^26^. The graft was lifted after osteotomy was performed across all borders to the cancellous portion. Also, the strut bones were harvested at a depth of at least 15 mm to ensure that they would have sufficient thickness. If the strut bone is harvested with a relatively thin thickness compared to its length, it may be fractured during the harvest. In addition, harvesting with sufficient thickness can increase the osteogenic property of the strut bone by containing more cancellous bone inside.

**Figure 2:**
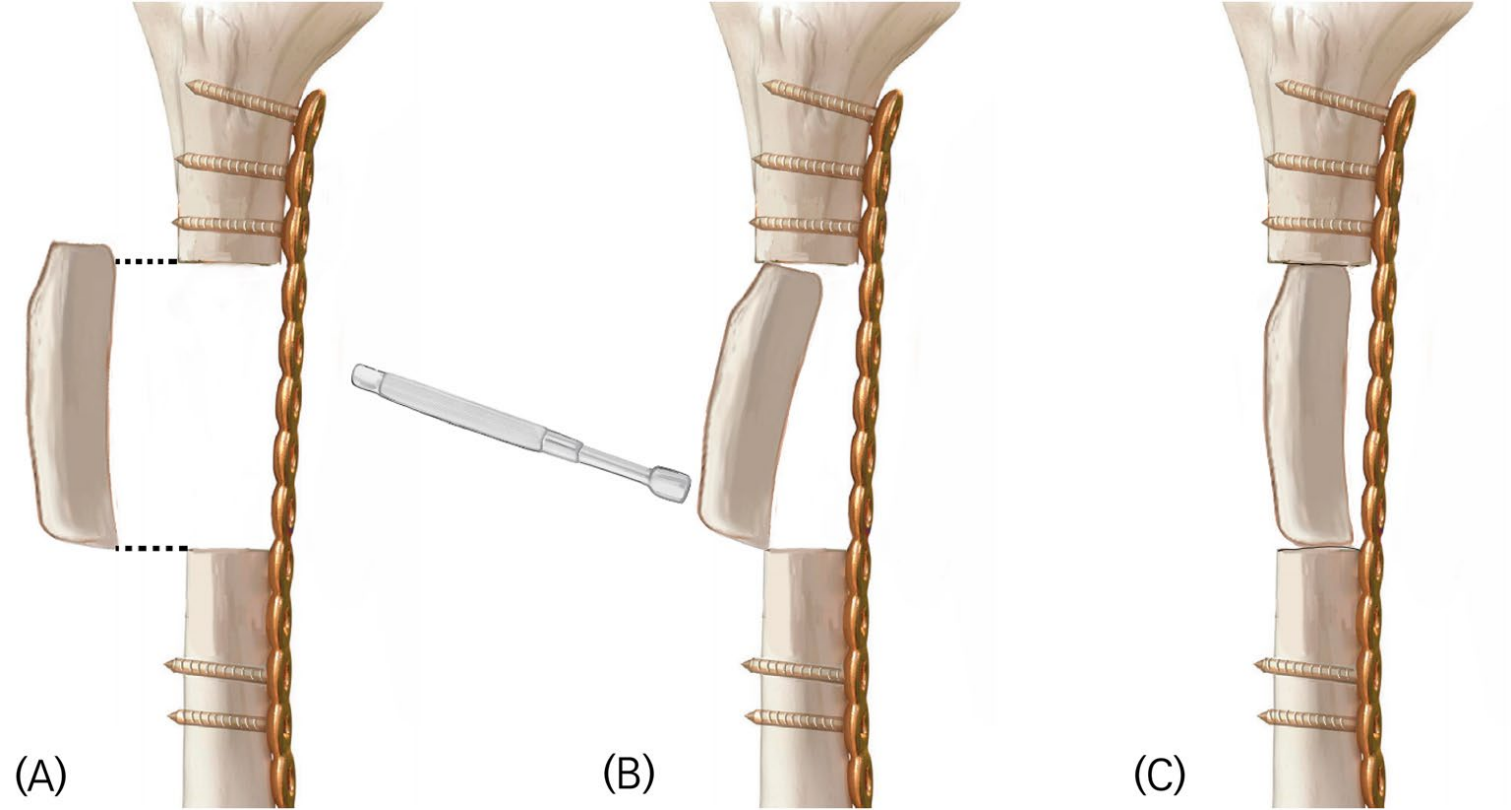
Schematic illustration of the autogenous iliac strut bone grafting technique. (**A**) The strut bone was harvested about 0.5 cm longer than the longitudinal length of the defect. (**B**) The strut bone was placed in the optimal position on the defect and then carefully struck with a mallet to press-fit the strut into the defect. (**C**) Sufficient compressive force was applied to both ends of the strut bone to promote union of the graft.

The strut bone was harvested at least 0.5 cm longer than the longitudinal length of the bone defect, and it was carefully press-fitted within the defect using a mallet in an optimized position to apply sufficient compressive force on the contact surface (Fig. 2). For the strut bone graft, cancellous bone from the same donor site was grafted at each end and middle of the strut. No additional cancellous bone was harvested from another site to minimize donor site morbidity, and no bone substitute was used during either the strut or cancellous bone grafting.

**Figure 3:**
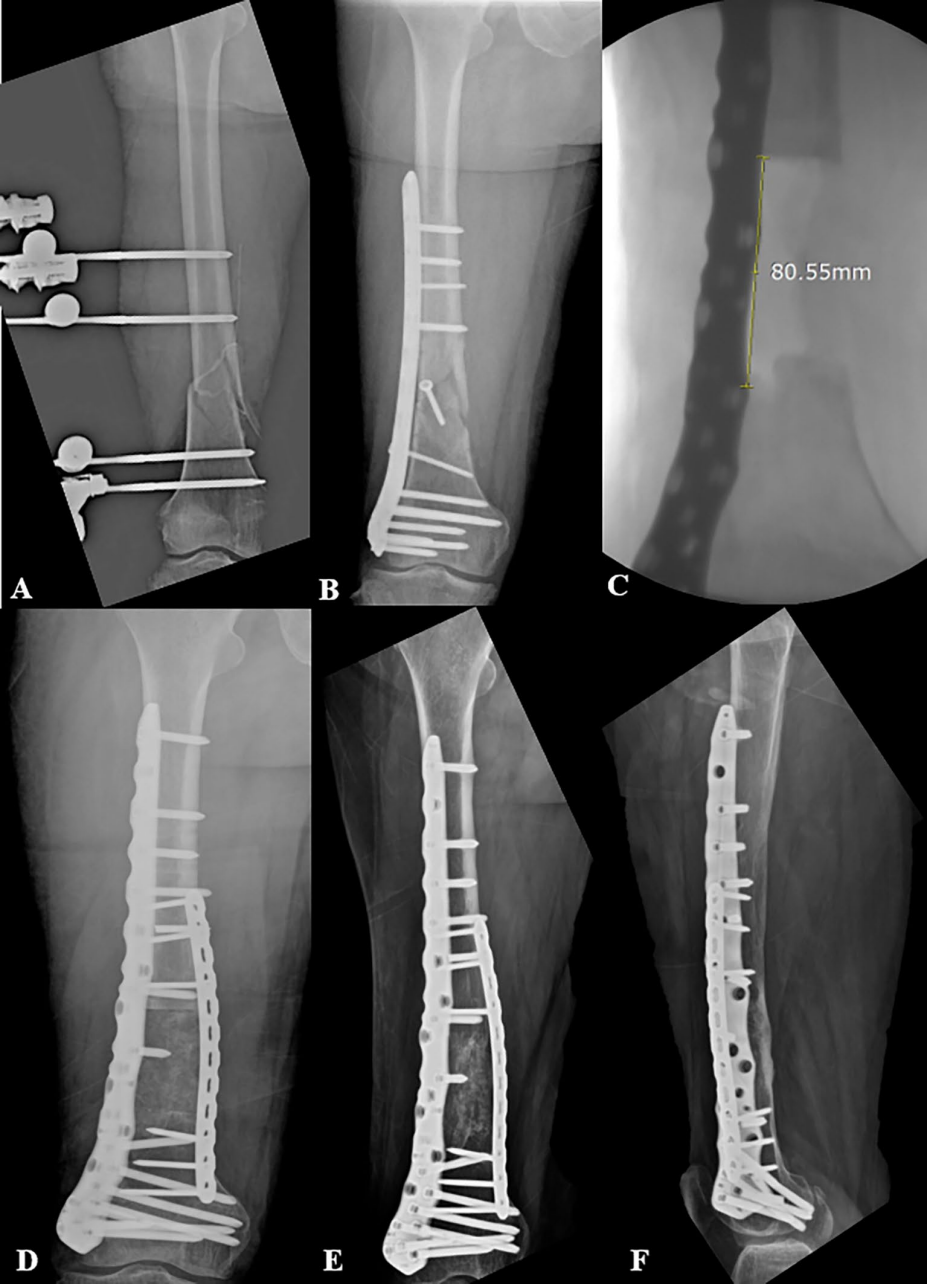
(**A**), (**B**) A 64-year-old female patient had a Gustilo-Anderson type III B open fracture of the distal 1/3 femur due to a pedestrian traffic accident. Initial management with temporary external fixation, followed by open reduction and internal fixation was performed at a local clinic. Six months after the surgery, no callus formation was observed. After examination, osteomyelitis was diagnosed, and the patient was referred to our hospital for further management. (**C**), (**D**) Serial debridement was performed until the infection was controlled. After infection control, the iliac strut bone was grafted onto the defect area with additional screw and plate fixation. The bone defect was measured to be 8.0 cm. (**E**), (**F**) Anteroposterior and lateral plain radiographs taken 24 months postoperatively. The patient achieved an uneventful complete bone union.

**Figure 4:**
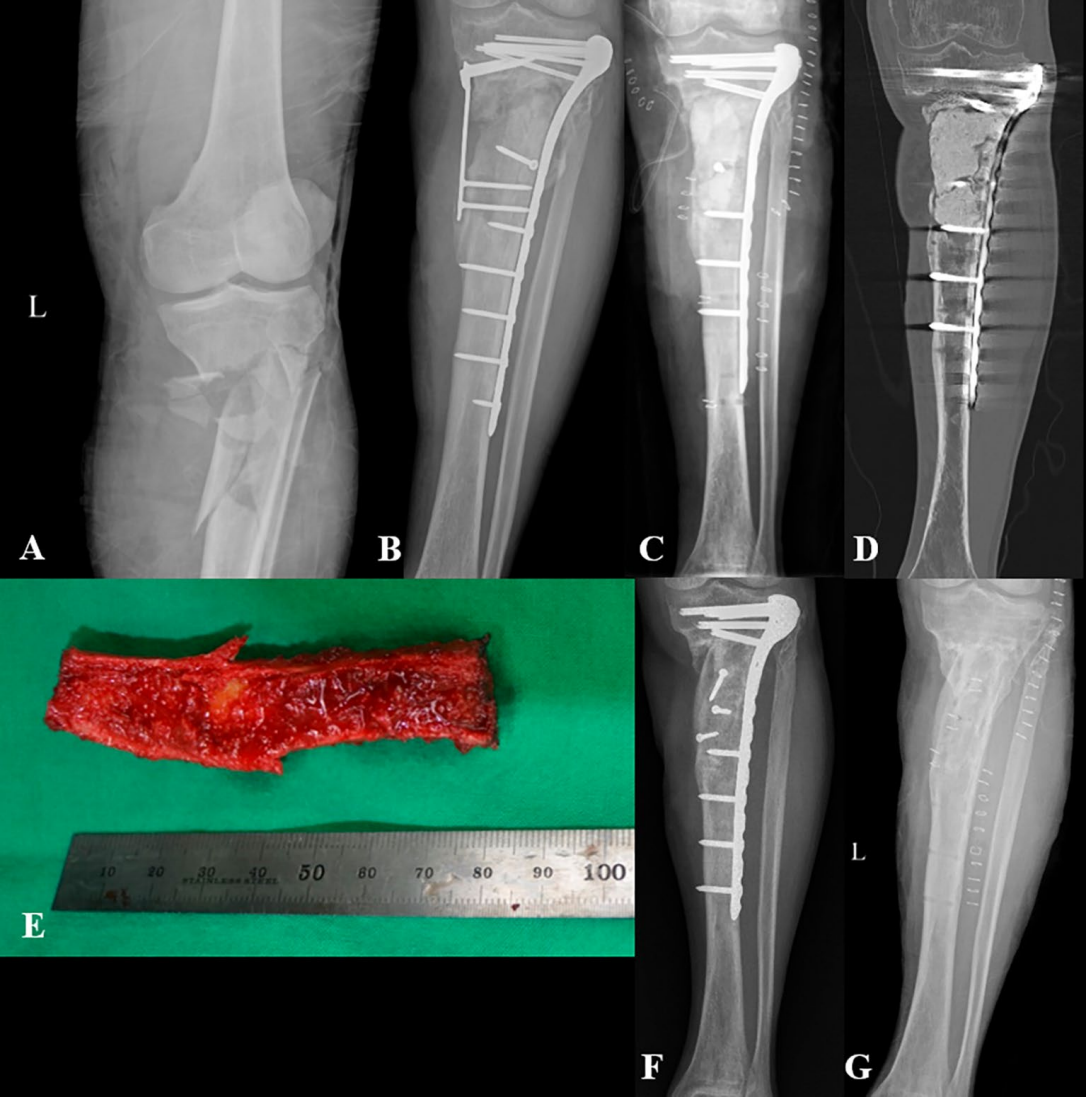
(**A**) 49-year-old male patient had a Gustilo-Anderson type III B open fracture of the left proximal tibia due to a motorcycle accident. (**B**) The fracture was treated with open reduction and internal fixation after debridement, and a rotation flap was performed for the skin defect. (**C**) At 9 months after surgery, surgical site infection was diagnosed, and antibiotic-loaded bone cement was inserted with plate fixation after serial debridement. (**D**) A coronal computed tomography image shows the cement spacer inserted into the bone defect. The bone defect length was measured to be 7.5 cm. (**E)**, (**F**) Eight weeks after the insertion of the cement spacer, the infection resolved, and autogenous iliac bone grafting was performed. An iliac strut bone was harvested 1 cm longer than the bone defect. Note that there is sufficient cancellous portion contained within the strut bone. (**G**) The patient achieved a complete bone union without complications.

The strut bone could be harvested up to 9–10 cm owing to the anatomical curvature of the iliac crest (Figs. 3 and 4). After harvesting the bone graft, bone wax was applied to the donor site to control bleeding, and a drainage tube was placed. For wound closure, the overlying muscles were tightly closed over the iliac crest, and a layer-by-layer suture was performed. All AIBG procedures were performed by a single orthopedic trauma surgeon. Soft tissue management, including flap surgery, was performed by a single plastic surgeon. In cases of infections, a two-stage surgery using the induced membrane technique (Masquelet Technique) was performed^27^.

### Postoperative rehabilitation and outpatient follow-up

For the postoperative rehabilitation protocol, active range-of-motion exercises were initiated the day after the surgery. Partial weight bearing using crutches was started 2–4 weeks after surgery for patients that underwent nail fixation and 6–8 weeks for patients that underwent plate fixation. Weight-bearing was progressively increased along the patient's pain-tolerable range. If the patient was judged to be able to walk independently, the walking aid was removed, and full weight bearing was permitted. Outpatient follow-ups were scheduled at 1, 2, 3, 6, 9, and 12 months, and the follow-up interval after 12 months was 6 months. Simple radiographs were obtained to assess the bone union and time to union at the graft site.

### Statistical analysis

Statistical analyses were performed using SPSS (version 27.0; SPSS Inc.). The power analysis was performed based on previous studies to estimate the sample size required for the study^10,28^. With an alpha level of 0.05, a minimum of 32 subjects was required to achieve a minimum statistical power of 0.80. Therefore, the minimum number of patients in each group was set at 16. Normality tests were performed for continuous variables using the Shapiro–Wilk test. Independent *t*-tests were used when normality was satisfied, and the Mann–Whitney U test was used for nonparametric data. Categorical variables were compared and analyzed using the chi-squared or Fisher's exact test. Results are presented as mean ± standard deviation. Statistical significance was set at *p* < 0.05. Union, time to union, and 6-month RUST score were the primary outcome measures, and the secondary outcomes included complications and reoperation.

### Ethical approval and informed consent

This retrospective study was performed in accordance with the principles of the Declaration of Helsinki and was approved by the Institutional Review Board of Hanyang University Hospital (Approval no.: 2021-05-039). The requirement for informed consent was waived by the Institutional Review Board of Hanyang University Hospital due to the retrospective nature of the study.

## Results

Of the 61 enrolled patients during the study period, one was excluded due to the age being < 18 years and 10 were excluded due to a postoperative follow-up period of < 1 year. Finally, 50 patients were included in this study. The mean age of the patients was 50.0 ± 16.3 (range: 19–78 years). The study population comprised 37 men (74%) and 13 women (26%); there were six patients with diabetes (12%) and 17 smokers (34%). The mean follow-up period was 25.6 ± 19.6 months (range: 12–104 months). The causes of bone defects were trauma in 11 patients (22%), infection in 17 patients (34%), and nonunion in 22 patients (44%). All nonunion cases were classified as atrophic. The average bone defect length and defect volume for the entire study population was 4.1 ± 2.2 cm (range: 1.2–9.7 cm) and 19.7 ± 10.0 cm^3^ (range: 5.0–42.4 cm^3^), respectively. Except for one trauma case each in Groups S and C, the type of bone defect in all cases was segmental.

Group S included 16 patients with an average age of 55.6 ± 15.7 years (10 men and 6 women) (Table 1). In Group S, two patients had diabetes (12.5%), and four were smokers (25.0%). The mean follow-up period was 25.7 ± 21.6 months. The causes of bone defects were trauma in five patients (31.2%), infection in seven patients (43.8%), and nonunion in four patients (25.0%). Bone defects were located in the femur in three patients (18.8%) and the tibia in 13 (81.2%). The bone defect area was at the diaphysis in 13 patients (81.2%) and at the metaphysis in three patients (18.8%). The mean length of the bone defect was 6.8 ± 1.2 cm (range: 5.1–9.7 cm), and mean defect volume was 31.4 ± 6.0 cm^3^ (range: 20.7–42.4 cm^3^). There were four open fractures, all of which were G-A III B. Fixation using a plate was performed in 14 cases (87.5%), and bone grafting alone with retention of the existing implant was performed in two cases (12.5%). Flap surgery was performed in 11 cases (68.8%); four open fractures and seven cases of infection. Union was achieved in all patients in Group S, and the average time to union was 6.7 ± 1.6 months. Complications occurred in four patients (25.0%). All complications occurred at the recipient site, with three cases of flap dehiscence and one case of superficial infection. Flap dehiscence was assessed by a plastic surgeon. For superficial infections, intravenous antibiotics were administered, and they resolved without surgery. Transient sensory changes in the donor site were present in one patient but resolved completely after 1 month and were not regarded as complications. No reoperation due to nonunion or metal failure was performed in Group S (Table 2).

**Table 1:** Patient demographics and clinical characteristics regarding Groups S and C.

	Group S (n = 16)	Group C (n = 34)	*P*-value
Age (years)	55.6 ± 15.7	47.4 ± 16.2	0.098
Sex (male: female)	10:6	27:7	0.301
DM (%)	2 (12.5)	4 (11.8)	1.000
Smoking (%)	4 (25.0)	13 (38.2)	0.357
Follow-up period (month)	25.7 ± 21.6	25.6 ± 19.0	0.305
Cause (%)			0.173
Trauma	5 (31.3)	6 (17.6)	
Infection	7 (43.8)	10 (29.4)	
Nonunion	4 (25.0)	18 (52.9)	
Location (%)			0.118
Femur	3 (18.8)	14 (41.2)	
Tibia	13 (81.3)	20 (58.8)	
Area (%)			0.501
Diaphysis	13 (81.3)	23 (67.6)	
Metaphysis	3 (18.8)	11 (32.4)	
Bone defect (cm)	6.8 ± 1.2	2.8 ± 1.1	**< 0.001**
Defect volume (cm^3^)	31.4 ± 6.0	14.1 ± 5.8	**< 0.001**
Type of bone defect (%)			0.542
Segmental	15 (93.8)	33 (97.1)	
Partial	1 (6.2)	1 (2.9)	
Open fracutre (%)	4/5 (80.0)	5/6 (83.3)	1.000
G-A			0.444
3A	0	2	
3B	4	3	
Fixation method (%)			0.661
Plate	14 (87.5)	28 (82.4)	
IM nail	0 (0)	2 (5.9)	
Plate and IM nail	0 (0)	1 (2.9)	
Retain previous implants (BG only)	2 (12.5)	3 (8.8)	
Flap coverage (%)	11 (68.8)	9 (26.5)	**0.004**
Timing of flap			0.653
Prior to BG	4	5	
Simultaneously with BG	7	4	

*Group S*, Strut iliac bone graft group; *Group C*, cancellous iliac bone graft group; *DM*, diabetes mellitus; *G-A*, Gustilo-Anderson classification; *IM*, intramedullary; *BG*, bone graft.

Significant *P*-values are highlighted in bold.

**Table 2:** Information regarding patients with complications.

No	Sex	Age (year)	F/U (months)	Cause	Defect area, location	Defect size (cm)	Autograft type	Complication	Reoperation
1	Male	52	15	Trauma	Tibia Metaphysis	2.5	Cancellous	Superficial infection	No
2	Male	41	18	Trauma	Tibia Diaphysis	5.8	Strut	Flap dehiscence	No
3	Male	68	61	Nonunion	Tibia Metaphysis	1.6	Cancellous	Delayed wound healing	No
4	Male	59	21	Infection	Tibia Diaphysis	4.3	Cancellous	Implant failure	Yes
5	Female	49	24	Trauma	Tibia Metaphysis	5.7	Strut	Flap dehiscence	No
6	Male	61	30	Nonunion	Femur Metaphysis	1.8	Cancellous	Superficial infection	No
7	Female	68	14	Nonunion	Tibia Diaphysis	2.7	Cancellous	Delayed wound healing	No
8	Male	58	24	Infection	Tibia Diaphysis	6.7	Strut	Superficial infection	No
9	Female	67	36	Infection	Femur Diaphysis	5.1	Strut	Flap dehiscence	No

*F/U*, Follow-up.

Group C consisted of 34 patients with an average age of 47.4 ± 16.2 years. There were 27 men and seven women; four had diabetes, and 13 were smokers. Nonunion was the most common cause of bone defect in 18 patients (52.9%). The average bone defect length and defect volume were 2.8 ± 1.1 cm (range: 1.2–4.9 cm) and 14.1 ± 5.8 cm^3^ (range: 5.0–29.7 cm^3^), respectively, and open fractures were observed in five cases (Table 1). Complications occurred in five cases (14.7%) in Group C, including two of superficial infections in the recipient area, two of delayed wound healing, and one of implant failure. No donor-site complications were observed. Both superficial infections and delayed wound healing were resolved without additional surgery. Reoperation was performed in one case of implant failure (Table 2).

Comparative analysis was performed between Groups S and C. There were no significant differences in age, sex, diabetes mellitus status, smoking status, and follow-up period between the two groups (Table 1). There were no differences between the two groups in terms of the cause, location, area, and type of the bone defect. Moreover, the rate of open fractures, distribution of G-A types, fixation method, and timing of the flap operation did not differ significantly between the two groups (Table 1). There was no difference in the use of the induced membrane technique between the two groups (*p* = 0.318). There were significant differences in the bone defect length (*p* < 0.001), defect volume (*p* < 0.001), and rate of flap coverage (*p* = 0.004) (Table 1).

**Figure 5:**
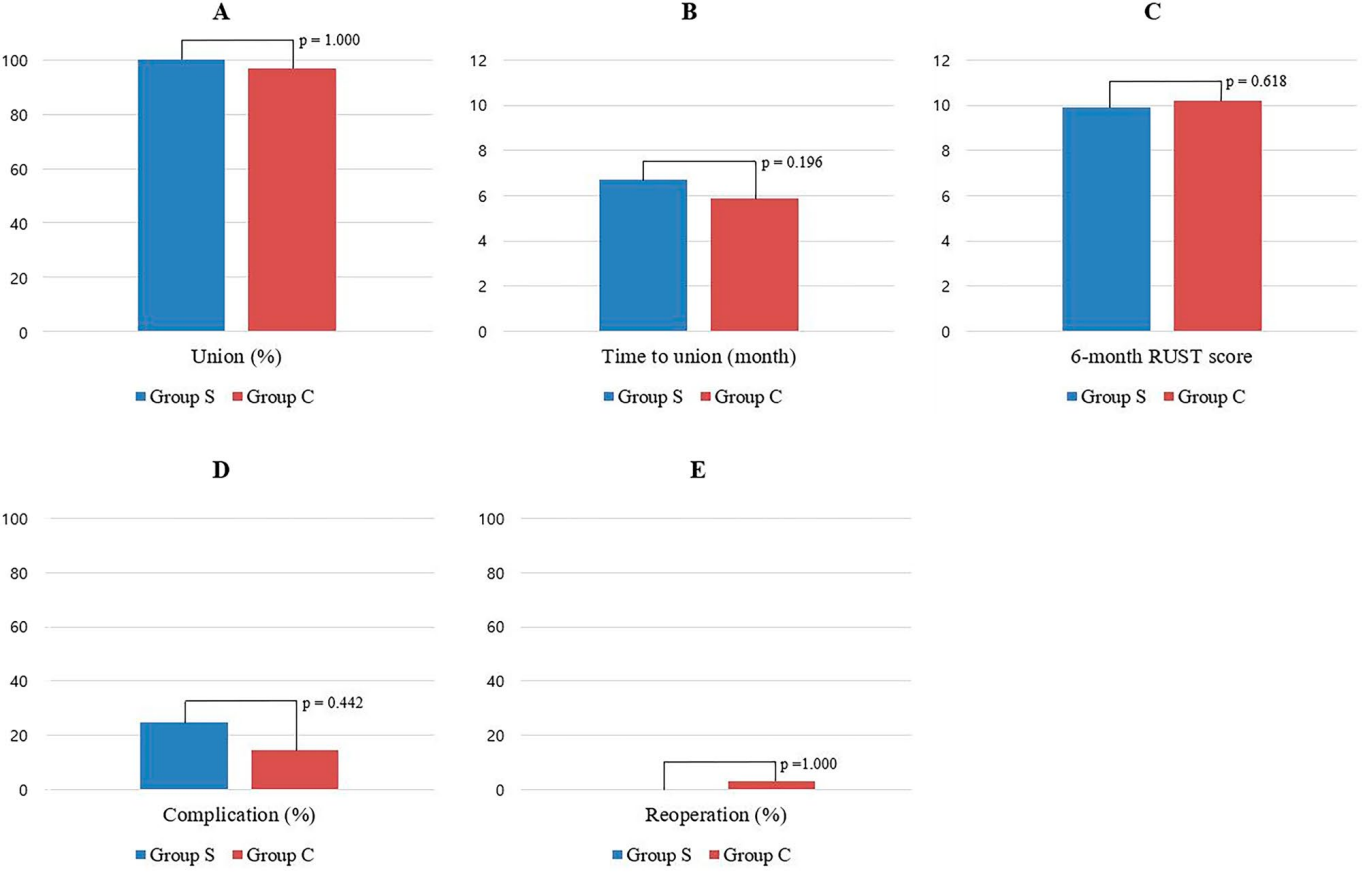
Comparison of primary and secondary outcomes between Group S and Group C. (**A**) Union. (**B**) Time to union. (**C**) Postoperative 6-month Radiographic Union Scale of Tibial Fractures (RUST) score. (**D**) Complication. (**E**) Reoperation.

For outcome measures, there was no significant difference in the union (Group S: 100.0% vs. Group C: 97.1%, *p* = 1.000) and the time to union (Group S: 6.7 ± 1.6 months vs. Group C: 5.9 ± 2.2 months, *p* = 0.196). The 6-month postoperative RUST score was 9.9 ± 1.6 in Group S and 10.2 ± 1.6 in Group C, which was not significantly different (*p* = 0.618). Moreover, there were no significant differences in the complication rate (*p* = 0.442) and reoperation rate (*p* = 1.000) between the two groups (Fig. 5).

A subgroup analysis was performed in each group to determine if there was a difference in outcome depending on the use of the induced membrane (two-staged) technique. In Group S, there was no difference in the union rate (two-stage: 100% vs. single-stage: 100%, *p* = 1.000), union time (two-stage: 6.6 ± 1.4 months vs. single-stage: 6.8 ± 1.9 months, *p* = 0.758), 6-month RUST score (two-stage: 10.9 ± 1.5 vs. single-stage: 9.7 ± 1.5, *p* = 0.142), complications (*p* = 1.000), and reoperation rate (*p* = 1.000) between two-staged and single-staged procedure. Group C also showed no difference in union rate (*p* = 0.294), union time (*p* = 0.956), 6-month RUST score (*p* = 0.838), complications (*p* = 1.000), and reoperation rate (*p* = 0.294).

## Discussion

This is the first study to evaluate the outcomes of iliac strut bone grafting in the lower extremities. This study investigated the results and complications of autogenous iliac strut bone grafting for bone defects ≥ 5 cm in the lower extremities. The results of this study demonstrated that the use of iliac strut bone grafts for bone defects ≥ 5 cm showed no significant differences regarding the union rate, time to union, complications, and reoperation rates compared to the use of autogenous iliac cancellous bone grafts for bone defects < 5 cm, suggesting that iliac strut bone graft in large bone defects is comparable in effectiveness to standard of care in small bone defects.

AIBGs are rich in angiogenic factors, colony-forming cells, and progenitor cells that are involved in bone union^29,30^. Despite the advantages, previous studies did not recommend the use of autogenous iliac cancellous bone grafts for bone defects ≥ 5 cm, as there were concerns about partial resorption of the grafted cancellous bone and weakening at the graft site due to creeping substitution (Table 3)^3,4,6–8^. In a study of 23 patients with a mean defect size of 8.5 cm in tibia, the union rate after conventional cancellous bone grafting was 74%, with complications of infection occurring in 6 patients (26.1%)^6^. A study of 10 patients with open tibia fractures with a mean defect of 5 cm reported a 40% union rate after cancellous bone grafting, and a study of 16 patients with a mean defect of 4.4 cm in the lower extremity reported a 75% union rate^7,8^. Previous studies have recommended distraction osteogenesis or vascularized bone grafting for large bone defect exceeding 5 cm^31,32^. Both methods have shown good results in the management of large bone defects. However, several drawbacks of distraction osteogenesis techniques have been reported, such as a long treatment period, patient discomfort, bone necrosis due to infection, local stress at the pin site, joint contracture, and nerve damage^33,34^. In particular, it has been reported that the risk of re-fracture of the transported bone can be high when distraction osteogenesis is performed greater than 8 cm^34^. Vascularized bone grafts are primarily harvested from the fibula. This procedure requires special expertise and a long operation time. Moreover, donor site morbidity is usually high because of extensive dissection. They also do not provide sufficient mechanical stability because of the small cross-sectional area of the fibula, which leads to re-fracture, and long-term protection is required^35,36^. The bioactive-induced membrane technique (Masquelet Technique) is a well-known method for treating large bone defects, and several favorable results have been reported^37^. Despite the many advantages of the induced membrane technique, it has been reported that bone integration is slow, and mechanical failure may occur^9^. In this study, single-stage surgery was performed in most cases, and two-step surgery using the induced membrane technique was performed in cases of infections^27^. The subgroup analysis in Group S of our study showed no difference in outcomes between the induced membrane technique and the single-stage procedure. A study comparing the induced membrane technique to primary bone graft in segmental bone defects also found no difference in union rates or complications between the two methods^38^. This suggests that combining patients who underwent the induced membrane technique with those who underwent primary bone grafting may provide more statistical power without increasing the heterogeneity of the study population. In Group S, bone union was achieved without mechanical failure in all patients, suggesting that the strut graft can be effectively used in conjunction with the induced membrane technique.

**Table 3:** Studies reporting results of single-stage cancellous bone grafting in large bone defects.

Authors	No. of cases	Mean defect size (cm)	Union (%)	Reoperation	Complication
Cierny III et al.^6^	23	8.5	74	Not reported	6 (Infection)
Watson et al.^7^	10	5	40	6 (Additional BG)	8 (Malunion)
Pelissier et al.^8^	16	4.4	75	5 (Additional BG)	2 (Flap necrosis)

BG: Bone graft.

The strut AIBG technique also has the advantage of being a simple procedure, providing a relatively large cross-sectional area of grafted bone and initial stability. The iliac strut grafts used in our study were harvested at a depth of at least 15 mm to ensure that sufficient cancellous bone was included within the strut to overcome the disadvantages of cortical bone grafts with poor osteogenic properties. Additional cancellous bone was also grafted at both ends of the strut to prevent slow revascularization and graft weakness that can occur in the early stages of grafting. The major cause of the poor results with cancellous bone grafting in large segmental bone defects is that the grafted bone is readily dispersed and resorbed by movement of surrounding muscles and tendons during postoperative rehabilitation^39,40^. Several studies have reported high rates of union using techniques that wrap around the grafted cancellous bone and hold it in place^41,42^. The strut bone used in our study also acted to wrap the cancellous bone inside, which may have contributed to a more favorable union rate. In Group S, bone union was achieved in all cases, and no reoperation due to union problems or implant failure was performed. These results indicate good mechanical stability of strut AIBGs.

One of the major drawbacks of AIBG is complications at the donor site. Chronic pain, hematoma, infection, and nerve injury were reported as the main complications^1,43–45^. However, the incidence of donor site complications was low in our study. Only one case of transient sensory change was observed. The lower complication rate can be attributed to strict adherence to modified surgical techniques to reduce soft tissue dissection, such as preserving the inguinal ligament, stopping the incision 3 cm posterior to the ASIS to prevent injury to the lateral femoral cutaneous nerve^23^, making a tight layer-by-layer suture with the overlying muscles and fascia in place after harvest, and achieving thorough hemostasis by applying bone wax at the harvest site^2,46^. In a study of complications after AIBG with a mean follow-up of 4.5 years, all patients reported no pain at final follow-up^47^. A retrospective study of 235 patients reported no complaints of donor site pain 6 weeks after AIBG, and only 2 patients (0.85%) developed hematoma^18^. In a meta-analysis study comparing complications of AIBG with autogenous bone grafting using a reamer irrigator aspirator (RIA), the rate of hematoma after AIBG was 1.4%, which was not significantly different from 0.9% of RIA (*p* = 0.4)^44^. A retrospective study of 42 patients reported no infection after AIBG and no donor site sensory changes were observed, and another study also reported that there was no infection after AIBG, and 52.3% of patients complained of sensory disturbances at 1 week postoperatively, but all resolved completely by 6 weeks postoperatively^18,48^. A prospective study of 92 patients who underwent AIBG reported that donor site sensory changes were observed in 8% of patients at the first postoperative outpatient visit, but resolved completely by 3 months postoperatively^19^. The very low rate of donor site complications observed in our study is consistent with the aforementioned recent studies. This demonstrates that the AIBG technique is a safer technique than previously known and suggests that autogenous iliac strut bone grafting can be performed without complications if it is performed cautiously.

Because of the anatomical curve of the iliac crest and the lateral femoral cutaneous nerve around the ASIS, the maximum length that could be harvested in the form of a strut was 9–10 cm. Strut AIBG technique is indicated for lower extremity bone defects 5–10 cm in length caused by infection, trauma, or nonunion. For bone defects greater than 9–10 cm, other procedures should be implemented. In addition, an important point when performing strut AIBG is that sufficient compressive force should be applied at the contact surface between the strut and the recipient bone. Sufficient compressive force limits the movement of the strut graft and allows it to unite uneventfully. If the compressive force is insufficient, nonunion will eventually occur at the end of the strut graft, and the grafted bone will be resorbed. Therefore, as we performed in this study, applying a sufficient compressive force to both ends of the strut graft is the key to a successful result. The strut graft was not used with IM nails in this study because the contact surface between the strut and host bone was small due to the nail being inside the canal. Further research is needed on the use of strut AIBG with IM nail.

According to a recent meta-analysis investigating the union rate after autogenous bone grafting, there was no direct correlation between the size of the bone defect and the rate of bone union^49^. It has been reported that the decision should be made comprehensively considering the patient's expectations, the surgeon's experience, and soft tissue condition rather than considering only the size of the bone defect when performing autogenous bone grafting^49^. Based on previous reports and our study, if strut AIBG is performed in an appropriate patient, the iliac strut graft can be effectively used to reconstruct the bone defect while compensating for the shortcomings of existing methods in the case of a large bone defect of up to 9–10 cm.

This study had some limitations. We compared the use of strut grafts for bone defects ≥ 5 cm and with the use of cancellous grafts for bone defects < 5 cm. Comparing cancellous AIBG with strut AIBG in bone defects ≥ 5 cm may have minimized heterogeneity, but study of such design may raise ethical issues and harm patients. We thus compared strut bone graft with cancellous bone graft in bone defects < 5 cm, which is the standard of care, to determine if strut AIBG is as effective as the standard of care without compromising the ethics of the study. Second, the type of graft was selected by the surgeon, and there may have been a risk of selection bias. However, this bias could be minimized by determining the graft type based on the size of the bone defect. In addition, since the surgeries were performed by a single surgeon at a single institution, surgical bias was minimal, and all patients were followed up for more than 1 year. Another limitation of the study was the relatively small number of patients included, although data was collected over a nine-year period. Future studies with more patients are needed to further elucidate the effectiveness of strut grafts in the induced membrane technique.

## Conclusions

Autologous iliac strut bone grafting is a safe and efficient method compared to autogenous iliac cancellous bone grafting and is expected to be effectively applied to bone defects larger than 5 cm in the lower extremities. Still, additional studies involving more patients treated with autogenous iliac strut bone grafts are need.

### Supplementary Information

Supplementary Information.

## Data Availability

All data analyzed in this study are included in this published article and its supplementary information file.
